# Anti-CD154 mAb and Rapamycin Induce T Regulatory Cell Mediated Tolerance in Rat-to-Mouse Islet Transplantation

**DOI:** 10.1371/journal.pone.0010352

**Published:** 2010-04-26

**Authors:** Yannick D. Muller, Gang Mai, Philippe Morel, Véronique Serre-Beinier, Carmen Gonelle-Gispert, Gisella Puga Yung, Driss Ehirchiou, Jean-Christophe Wyss, Sinda Bigenzahn, Magali Irla, Christoph Heusser, Déla Golshayan, Jörg D. Seebach, Thomas Wekerle, Leo H. Bühler

**Affiliations:** 1 Surgical Research Unit, Department of Surgery, University Hospital Geneva, Geneva, Switzerland; 2 Service of Clinical Immunology and Allergology, Department of Internal Medicine, University Hospital and Medical Faculty, Geneva, Switzerland; 3 Hepato- Bilio-Pancreatic Department, West China Hospital, Si Chuan University, Chengdu, Si Chuan, People's Republic of China; 4 Transplantation Immunopathology Laboratory, Department of Medicine, University of Lausanne, Lausanne, Switzerland; 5 Division of Transplantation, Department of Surgery, Vienna General Hospital, Medical University of Vienna, Vienna, Austria; 6 University of Geneva Medical School, CMU, Geneva, Switzerland; 7 Novartis Institutes for Biomedical Research, Basel, Switzerland; New York University, United States of America

## Abstract

**Background:**

Anti-CD154 (MR1) monoclonal antibody (mAb) and rapamycin (RAPA) treatment both improve survival of rat-to-mouse islet xenograft. The present study investigated the effect of combined RAPA/MR1 treatment on rat-to-mouse islet xenograft survival and analyzed the role of CD4^+^CD25^+^Foxp3^+^ T regulatory cells (Treg) in the induction and maintenance of the ensuing tolerance.

**Methodology/Principal Findings:**

C57BL/6 mice were treated with MR1/RAPA and received additional monoclonal anti-IL2 mAb or anti CD25 mAb either early (0–28 d) or late (100–128 d) post-transplantation. Treg were characterised in the blood, spleen, draining lymph nodes and within the graft of tolerant and rejecting mice by flow cytometry and immunohistochemistry. Fourteen days of RAPA/MR1 combination therapy allowed indefinite islet graft survival in >80% of the mice. Additional administration of anti-IL-2 mAb or depleting anti-CD25 mAb at the time of transplantation resulted in rejection (100% and 89% respectively), whereas administration at 100 days post transplantation lead to lower rejection rates (25% and 40% respectively). Tolerant mice showed an increase of Treg within the graft and in draining lymph nodes early post transplantation, whereas 100 days post transplantation no significant increase of Treg was observed. Rejecting mice showed a transient increase of Treg in the xenograft and secondary lymphoid organs, which disappeared within 7 days after rejection.

**Conclusions/Significances:**

These results suggest a critical role for Treg in the induction phase of tolerance early after islet xenotransplantation. These encouraging data support the need of developing further Treg therapy for overcoming the species barrier in xenotransplantation.

## Introduction

The inhibition of co-stimulation (signal 2) and proliferation (signal 3) of T cell activation by co-stimulatory blockade and rapamycin (RAPA) induces peripheral tolerance to allografts [1]–[3]. In contrast to central tolerance where self-antigen specific T cells are depleted in the thymus, peripheral tolerance is achieved by various mechanisms including: apoptosis of activated T cells, T cell anergy, and active regulation by T regulatory cells (Treg) [4], [5]. CD4^+^CD25^+^Foxp3^+^ T cells remain currently the best characterized population of Treg in experimental transplantation (Tx) [6].

The variable role of Treg in the induction and maintenance of allograft tolerance has been described in numerous models. Induced Treg or *ex-vivo* generated antigen specific Treg have been shown to protect allografts from immune-mediated damage [7]–[9]. However, it is still poorly understood where and when tolerization through Treg takes place [10]. Moreover their potential role in xenogeneic models remains to be defined [11].

We previously reported that treatment with anti-CD154 mAb (MR1) significantly improved survival of rat islet xenograft in mice [12], [13]. Furthermore, RAPA has been shown to selectively promote expansion of Treg *in vitro* and to prevent allograft rejection *in vivo*. [14] [15], [16]. The aim of the present study was to investigate the effect of RAPA/MR1 combination therapy on long-term xenograft acceptance and to further analyze the mechanisms leading to tolerance, in particular the role of regulation by CD4^+^ IL-2-dependent CD25^+^Foxp3^+^ Treg.

## Materials and Methods

### Animals

C57BL/6 mice between 6–10 weeks old were used as recipients (Centre de Recherche et d'Elevage, Janvier, France). Adults Sprague-Dawley rats, approximately 300–350 grams of body weight, were used as islet donors (Janvier). Animals were maintained in conventional housing facilities. Experiments involving animals were performed in compliance with relevant laws according to Geneva veterinary authorities and were approved by the ethical committee of the Geneva University Medical School.

### Islet isolation and xenotransplantation

Recipient mice were made diabetic by single intraperitoneal (i.p.) injection of streptozotocin (Sigma, Buchs, Switzerland), 220 mg/kg between 72–96 hours prior transplantation. Blood sugar levels were monitored on regular intervals using a commercial kit (Precision Q.I.D, MediSence, Abbott, Bedford, MA). Only mice with blood sugar levels >17 mmol/L on two consecutive days were used for Tx. Rat pancreatic islets were isolated as previously described [12]. Minimum 300 islet equivalent were transplanted per mice under the left kidney capsule.

### Experimental design

Anti-CD154 monoclonal antibody (mAb) (MR1, hamster anti-mouse CD154 mAb (CD40L), Bio Express, West Lebanon, NH) diluted in PBS (Sigma) was administered i.p. at 0.5 mg per mouse on days 0, 2 and 4 post-Tx. The first dose, on day 0, was given 5 hours prior to Tx. Rapamycin (RAPA) (WYETH, Zug, Switzerland) diluted in distilled water was administered by oral gavage at 0.2 mg/kg on the first 3 days post-Tx, then every other day up to day 14. Anti-IL-2 mAb (S4B6-1) and anti-CD25 mAb (PC61), kind gifts from Novartis AG (Basel, Switzerland), were diluted in PBS and administered i.p. 0.5 mg for the first injection, then 0.3 mg twice weekly for four weeks respectively. Anti-IL-2 and anti-CD25 were started either at the time of Tx (early) or after 100 days (late). The following experimental groups were analyzed:

Group 1) Islet transplantation without further therapy (n = 34)

Group 2) RAPA therapy alone (n = 6)

Group 3) MR1 therapy alone (n = 9)

Group 4) Combination therapy RAPA/MR1 (n = 21)

Group 5) Combination therapy RAPA/MR1 with early anti-IL-2 injection (n = 10)

Group 6) Combination therapy RAPA/MR1 with late anti-IL-2 injection (n = 4)

Group 7) Combination therapy RAPA/MR1 with early anti-CD25 injection (n = 9)

Group 8) Combination therapy RAPA/MR1 with late anti-CD25 injection (n = 7)

### Graft survival follow-up

Islet graft function was determined by regular blood sugar determination (Precision Q.I.D). Blood sugar levels of <11 mmol on two consecutive days defined successful islet function. Blood sugar levels of >13 mmol on three consecutive days or a blood sugar level of >17 mmol defined graft rejection.

### Flow cytometry

CD4^+^CD25^+^Foxp3^+^ cells were considered as Treg and were stained according to the manufacturer's instructions (PE/APC anti-Foxp3 (FJK­16s), FITC anti-CD4 (RM4–5), APC anti-CD25 (PC61.5), eBioscience, San Diego, CA). For Group 7 and 8, animals receiving anti-CD25 mAb (PC 61.5), a non-crossreacting clone of anti-CD25 mAb APC was used to detect Treg by FACS (clone 3C7, Becton Dickinson, Franklin Lakes, NJ). Isotype control antibodies were purchased from Becton Dickinson (FITC rat IgG2a mAb, PE rat IgG2a, APC rat IgG1 mAb, APC rat IgG2b mAb, APC rat IgG2a).

Levels of Treg were measured in blood on days 5, 10, 20, 50 and 100 in mice of Group 1, 4, 5, 7. Moreover, spleen and para-aortic lymph nodes (paLN) were harvested for analysis of the percentage and total numbers of Treg at various time points. Group 1 animals were sacrificed either in the first 48 hours after rejection, or between 48 hours and 7 days after rejection. In Group 4, tolerant mice were sacrificed at days 20 and 100. Cell counts were performed using Beckman coulter Z series (Hialeah, FL). Flow cytometry acquisition was performed on FACScalibur (Becton Dickinson) and data analyzed using Flowjo software (Tree Star v. 8.7.3, Ashland, OR).

### Histopathology and immunohistology

Nephrectomy (graftectomy) was performed either early after rejection (<48 h) or between 48 h and 7days, or at day 20, 100 or 200 days post-islet Tx in tolerant recipients. Kidneys were either preserved in formol 10% or frozen at −80°C.

Paraffin: kidney samples were fixed in formol 10%, then embedded in paraffin and sectioned. Paraffin sections were used for hematoxylin and eosin (HE). For insulin staining, sections were incubated using guinea pig anti-porcine insulin antibody (DAKO A564, Denmark) and subsequently with goat anti-guinea pig Alexa 488-conjugated (Invitrogen, Basel, Switzerland). Sections were analyzed using a confocal microscope LSM510 meta (Zeiss Axiophot, Göttingen, Germany). For Foxp3 staining, sections were baked at 55°C for 60 minutes, cooled, then deparaffinized and rehydrated through graded alcohols to water. After antigen retrieval in heated Tris-EDTA-Tween buffer, endogenous peroxidase and biotin were blocked. The slides were then incubated with biotinylated anti-Foxp3 (clone FJK-16s, eBioscience) followed by Streptavidin/HRP (DAKO PO397) and liquid diaminobenzidine-tetrahydrochloride plus substrate (DakoCytomation), rinsed with water and counterstained with hematoxylin. Slides were analyzed under an axiocam microscope (Zeiss).

Cryostat: kidney samples were stored at −80°C as previously described [12]. Briefly serial frozen sections were stained for characterization of infiltrating cells with anti CD4 mAb and anti-CD8 mAb and anti mac-1 (Morphosys AbD Düsseldorf, Germany). The slides were examined by fluorescence microscopy (Zeiss).

### *In vitro* suppression assays

CD4^+^CD25^+^ Treg and CD4^+^CD25^−^ T cells were isolated from spleens of Group 1, Group 4 and naive mice by CD4 negative selection followed by CD25 positive selection using a CD4^+^CD25^+^ regulatory T cell isolation kit (Miltenyi Biotec, Bergish Gladbach, Germany). Treg and CD4^+^CD25^−^ T cell purities were greater than 90%. A total of 5×10^4^ CD4^+^CD25^−^ T cells isolated from naive mice were co-cultured with 1×10^5^ irradiated (3500 Rad) syngeneic splenocytes and anti-CD3e mAb (clone 145-2C11, eBioscience). Treg of Group 1, Group 4 or naive mice were then added in triplicates at different ratios (50×10^3^, 25×10^3^, 12.5×10^3^ and 6×10^3^). Alternatively for the xenogeneic suppression assays, a total of 0.4×10^6^ splenocytes isolated from naive mice were co-cultured with 0.6×10^6^ irradiated (3500 Rad) xenogeneic third party Lewis or donor Sprague Dawley splenocytes. 0.1×10^6^ Treg of Group 1, Group 4 were then added in triplicates to the cultures. On day 5, cells were pulsed with 1µCi^3^[H] of thymidine for 18 hours and then harvested. Results are expressed as CPM showing one representative experiment. In the xenogeneic suppression assays results are shown as the percentage of suppression and were calculated as:

100 * (CPM: naïve splenocytes and xenogeneic stimulators) – (CPM: naïve splenocytes, xenogeneic stimulators and Treg)/(CPM: naïve splenocytes and xenogeneic stimulators)

One representative experiment is shown from at least two separate experiments regrouping a total of 3 animals.

### Quantitative RT-PCR

CD4^+^CD25^+^ T cells total RNA were harvested from the spleen of rejector and tolerant recipient and prepared with Trizol. cDNA was synthesized with random hexamers and Superscript II reverse transcriptase (Invitrogen). PCR was performed with the iCycler iQ Real-Time PCR Detection System and iQ SYBR green Supermix (Bio-Rad).Results were quantified with a standard curve generated with serial dilutions of a reference cDNA preparation. GAPDH mRNA was used for normalization of Foxp3, IL-10 and TGF-beta1 mRNA expression. Primer sequences are listed in Table S1.

### Statistical analysis

Prism software was used for statistical analysis (GraphPad Software, San Diego California, USA). Survival curves were calculated by the Kaplan and Meier method and analyzed using Cox-Mantel test. MLR and *in vitro* suppression assays were analyzed using one way ANOVA test; Bonferroni's multiple comparison method was used as post test. Non parametric Kruskal Wallis test was used for analyzing the medians of Treg levels. A p-value inferior to 0.05 was considered statistically significant.

## Results

### Islet graft survival

**Figure 1:**
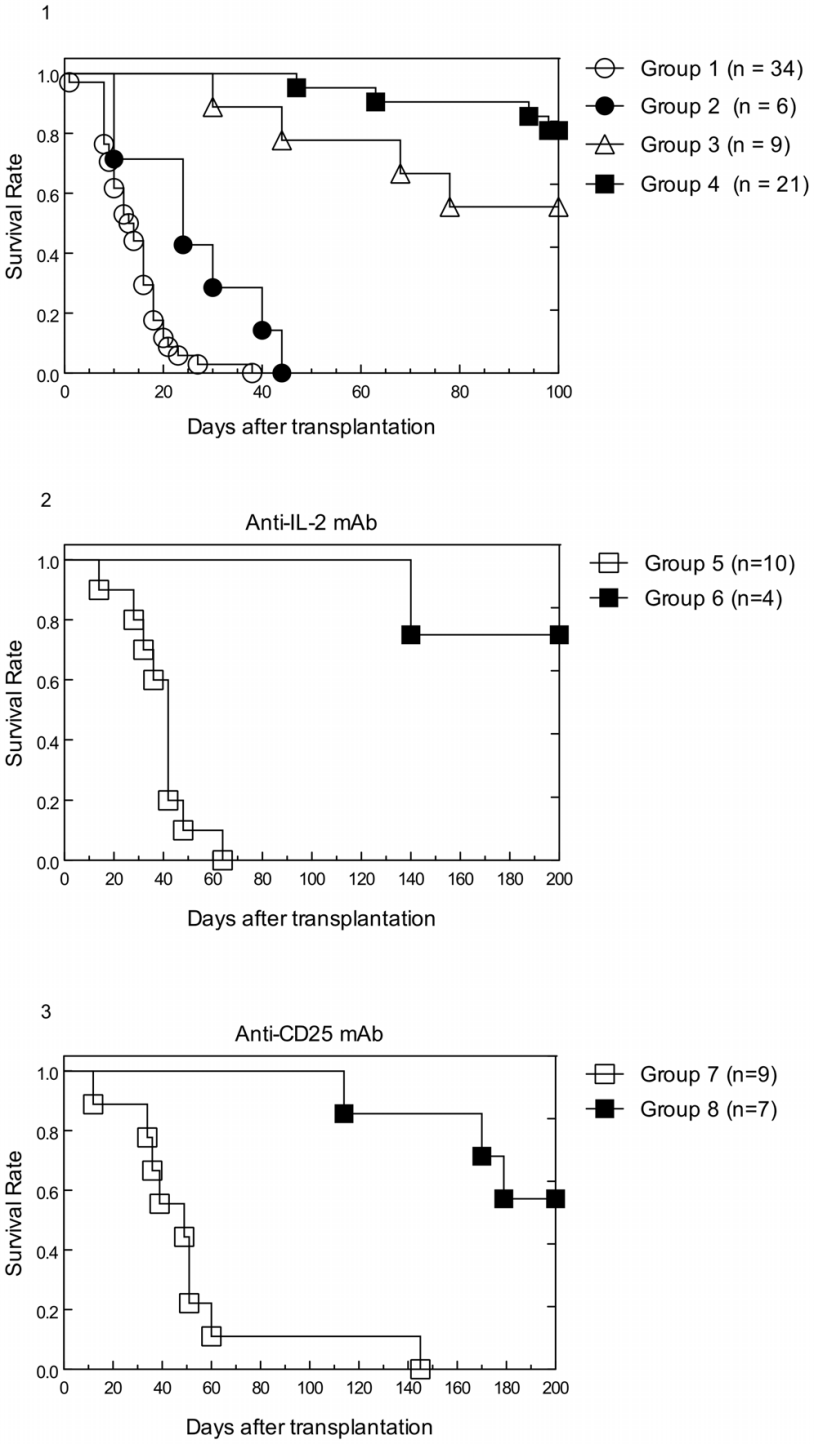
Graft survival curves of the experimental Groups. **A**: Islet survival groups in mice receiving no additional therapy (Group 1), rapamycin (RAPA) therapy alone (Group 2), anti-CD154 mAb (MR1) therapy alone (Group 3) and combination therapy of RAPA and MR1 (Group 4). Effect of anti-IL-2 (**B**): Exogenous anti IL-2 mAb administration at the time of islet transplantation (Tx) (Group 5) or 100 days post Tx (Group 6) in RAPA/MR1 treated mice. Effect of anti-CD25 (**C**): Exogenous anti CD25 mAb administration at the time of islet Tx (Group 7) or 100 days post Tx (Group 8) in RAPA/MR1 treated mice.

We first compared the effect of RAPA and MR1 on xenogeneic islet graft survival, given alone or in combination, as summarized in Figure 1A. Mice receiving no further therapy (n = 34) had a median graft survival (MGS) of 13.5 days (Group 1). RAPA therapy alone significantly prolonged rat islet survival to 24 days (p<0.01), but all grafts were still rejected (Group 2). MR1 therapy alone also significantly prolonged concordant islet xenograft survival, in 5 of 9 mice (55.6%, p<0.0037) accepting their graft over 100 days (Group 3). In Group 4, combination therapy with MR1 and RAPA for 14 days resulted in graft survival for >100 days in 17/21 (80.9%, p<0.001) recipients.

We then investigated the mechanism underlining long-term graft acceptance by administration of anti-IL-2 or anti-CD25 mAb either at the time of Tx or 100 days post-Tx. In Group 5, early administration of anti-IL-2 mAb also led to rejection in 10/10 mice (MGS 42 days). However, when anti-IL-2 mAb was given 100 days post-Tx, only 1 of 4 mice developed rejection at day 200 post-Tx (Group 6; Figure 1B). In Group 7, early administration of anti-CD25 mAb, led to rejection in 8/9 mice (MGS 49 days). However, when anti-CD25 mAb was given 100 days post-Tx, only 3 of 7 mice developed rejection at day 200 post-Tx (Group 8; Figure 1C). Late tolerant mice (200 days post-Tx) were shown to be hypo-responsive against donor antigen in contrast to rejecting or naive recipient in mixed lymphocyte reaction (Figure S1, Material and Methods S1).

### Levels of Treg in blood, spleen and para-aortic lymph nodes of tolerized mice vs rejecting mice

**Figure 2:**
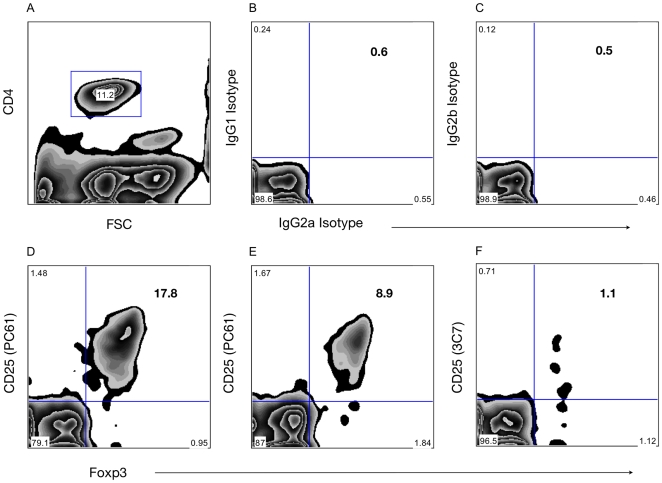
Representative analysis of CD4^+^CD25^+^Foxp3^+^ cells in blood of C57BL/6 mice. Percentages of CD25^+^Foxp3^+^ Treg gated in CD4^+^ T cell in the peripheral blood. Representative example of the gating in CD4^+^ T cells and the isotype controls used for CD25 (IgG1: pC61, IgG2b: 3C7) and Foxp3 (IgG2a) are shown in the upper panel (**A**, **B**, **C**). Representative analysis of CD25^+^Foxp3^+^ Treg in Group 1, i.e. islet transplantation without further therapy, at day 20 post-transplantation (**D**) in naive mice (**E**) and in Group 7, i.e early anti-CD25 mAb at day 20 post-transplantation (**F**).

In order to understand the possible role of Treg in the acceptance of islet xenograft their presence were investigated in the blood, lymph nodes and spleen of tolerant and rejecting mice. The percentages of CD25^+^Foxp3^+^ Treg within CD4^+^ T cells in 6 weeks old naive mice were as previously described (Figure 2A–B, 2E) [9], [17], [18].

**Figure 3:**
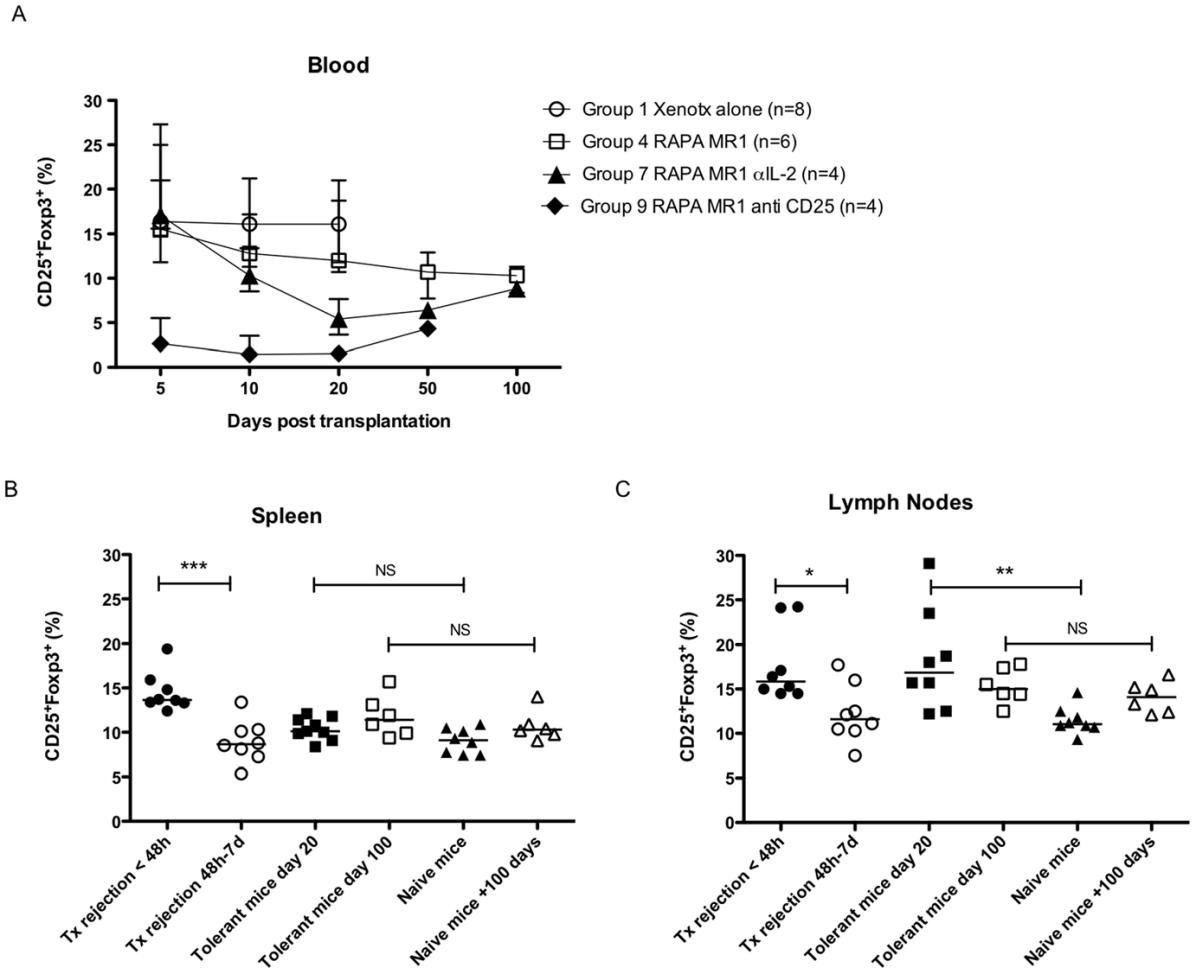
Flow cytometry analysis for Treg level in blood, spleen and para-aortic lymph nodes. **A**: Blood Treg levels over time in Group 1 (transplanted mice without further therapy), Group 4 (RAPA/MR1 treated mice), Group 5 (RAPA/MR1 treated mice and early administration of anti IL-2 mAb), and Group 7 (RAPA/MR1 treated mice and early administration of anti CD25 mAb). Treg levels in spleen (**B**) and para-aortic lymph nodes (**C**). In Group 1, mice were analyzed <48 hours of rejection and between >48 hours and 7 days of rejection. In Group 4 Treg levels were analyzed 20 days post transplantation. All results were calculated with median and range. Kruksal-Wallis test and Dunn's Multiple Comparison post Test was used. * P<0.05, ** P<0.01 and ***P<0.001 were considered significant.

In Group 1, blood analysis of transplanted mice without further therapy showed an increase of Treg from 9.8% at baseline to 16.4% at day 5 post-Tx and remained stable at day 20 post-Tx (16.1%) (Figures 2D, 3A). Xenograft-rejecting mice were characterized within the first 48 hours of rejection by a significant increase of Treg percentages in the spleen from 9% at baseline to 13.7% (Figure 3B) and in paLN from 11.5% to 15.9% (Figure 3C). Between 48 hours and 7 days after onset of rejection, the level of Treg decreased again to 8.7% in the spleen (Figure 3B) and to 11.1% in paLN (Figure 3C). Absolute numbers of Treg varied according to the percentage (data not shown). In summary, rejecting mice showed an increase of Treg in secondary lymphoid organs at the time of rejection, but after 48 hours to 7 days this increase of Treg has faded away.

In Group 4, blood analysis of tolerant mice showed an increase of the percentage of Treg from 9.8% at baseline to 14.8% at days 5 and to 12% 20 days post-Tx (Figure 3A). The percentage of Treg in the spleen of tolerant mice was comparable to naive mice of corresponding age (10.1% vs 9.1% at day 20, 11.4% vs 10.3% at day 100, p>0.05 Figure 3B). In contrast, tolerant mice had significantly higher levels of Treg in paLN at day 20 as compared to naïve mice (16.9% vs 11.5%, respectively p<0.01, Figure 3C). After 100 days, no difference was detected in paLN between tolerant mice and naive mice of corresponding age (15% vs 14.1%, Figure 3C). Absolute numbers of Treg varied according to the percentage (data not shown). Tolerant mice developed thus a transient increase in the percentage and number of Treg in paLN after tolerance induction 20 days post transplantation. This difference was however not detectable any more at 100 days when comparing tolerant to control naïve mice.

In Group 5, blood analysis of transplanted mice receiving RAPA/MR1 and early anti-IL-2, showed a decrease in Treg levels to 5.4% at day 20 post-Tx (Figure 3A). Group 7, transplanted mice receiving early anti-CD25, decreased Treg levels to 1.5% in blood at day 20 post-Tx (Figures 2C, 2F, 3A). As previously reported, both anti-CD25 mAb and anti-IL-2 mAb efficiently deplete Treg in the peripheral blood.

The surgical procedure itself (sham transplantation), combined with the injection of NaCl under the kidney capsule, had no effect on Treg percentage in the blood (data not shown). The effect of streptozotocin injected alone without further therapy was tested in our model. Interestingly, this procedure increased Treg levels at day 10 post-injection to 13.7% in blood, 13% in spleen and 17.7% in paLN. Theoretically, this increase could be related to the application of streptozotocin and/or the ensuing hyperglycemia. Thus, the respective effect of streptozotocin and hyperglycemia on Treg levels needs further investigation. All Groups of mice tended to restore blood Treg levels over 50 days.

### Histopathology and immunohistology of rat islet (graft) in the mouse kidney (recipient)

**Figure 4:**
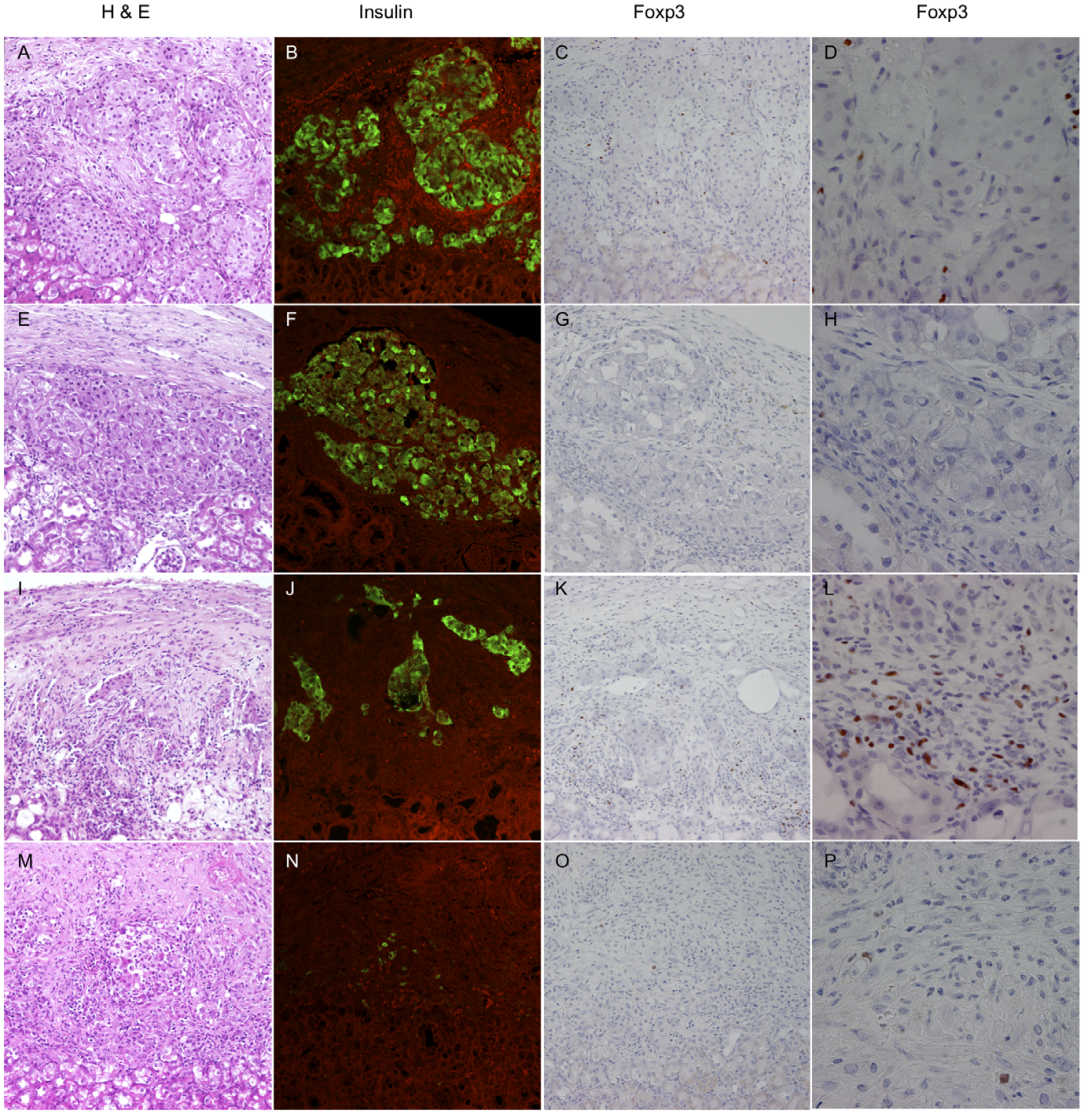
Immuno-histology of grafts in tolerant vs rejecting recipients. Graft staining of Group 4 (tolerant mice) at day 20 post-transplant (**A**–**D**), graft staining of Group 4 (tolerant mice) at day 100 post-transplant (**E**–**H**), graft staining of Group 1 (rejecting mice) within the first 48 hours of rejection (**I**–**L**) and graft staining of Group 1 (rejecting mice) between 48 hours and 7 days of rejection (**M**–**P**). **A**, **E**, **I**, **M**, Hematoxylin & eosin (magnification 200x), **B**, **F**, **J**, **N** Insulin immunofluorescence (green, magnification 200x), Foxp3 immunochemistry (brawn intranuclear staining) **C**, **D**, **G**, **K** Magnification 200x and **D**, **H**, **L**, **P** Magnification 600x.

The presence of Treg within the graft was analyzed by histology and immunohistological staining as shown in Figure 4. In RAPA/MR1 treated tolerant mice (Group 4), Hematoxilin and eosin (HE) analysis showed engraftment of islets under the kidney capsule at day 20 post-transplant with minimal cellular infiltration within the grafts (Figure 4A). Insulin staining confirmed well-preserved islets (Figure 4B) and Foxp3 positive cells were present within the graft (Figure 4C and D). After 100 days, islets of tolerant mice were still preserved and HE analysis showed minimal cellular infiltration of the grafts (Figure 4E), insulin staining confirmed the presence of islets (Figure 4F) but Foxp3 positive cells were detected only at very low frequency (Figure 4G–H).

In untreated mice (Group 1), HE analysis during early acute rejection (<48 h) showed strong mononuclear cellular infiltration (Figure 4I), insulin staining revealed islet disruption (Figure 4J) and Foxp3 positive cells were present in the graft (Figure 4K–L). HE staining of rejecting grafts between 48 hours and 7 days showed areas of necrosis and tissue remodeling (Figure 4M). Only rarely islets cells stained positive for insulin confirming the destruction of islets (Figure 4N). There were few/no Foxp3 positive cells suggesting that Treg disappeared after the destruction of the islets (Figure 4O–P).

**Figure 5:**
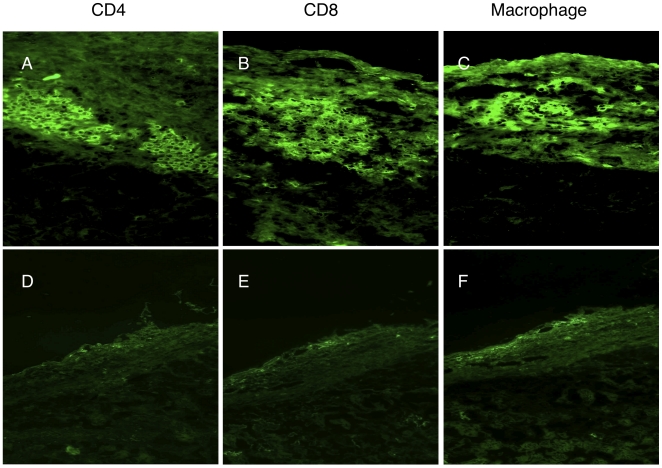
Cellular infiltration in rejecting and tolerant grafts. Cryostat sections were stained by anti-mouse CD4 (**A**–**D**), CD8 (**B**–**D**) and macrophage antibodies (**C**–**E**) in Group 1 (**A**–**B**–**C**) and Group 4 at 200 days post transplantation. In Group 1 of rejecting mice, immunohistology for cellular immune responses to concordant islet xenografts at time of rejection has detected mixed cellular infiltrates with presence of CD4+ (**A**), CD8+ (**B**), and macrophages (**C**). In Group 4 of tolerant mice, immunohistology for cellular immune responses to concordant islet xenografts of tolerant mice at 200 days post-transplantation detected only minimal cellular infiltrate with absence of CD4+ (**D**), CD8+ (**E**), and macrophages (**F**). (Magnification in **A**–**C** (200x), Magnification in **D**–**F**(100x)).

Cryosection analysis of rejecting grafts was characterized by cellular infiltration containing CD4+, CD8+ lymphocytes and macrophages (Figure 5A–C). A humoral response was also detected with IgG, IgM and C3 deposition (Figure S2). In contrast, RAPA/MR1 treated mice showed minimal cellular infiltration within the grafts 200 day post-Tx (Figure 5D–F) and neither immunoglobulin (IgG and IgM) nor complement deposition was observed (Figure S2).

### Treg of tolerant and rejecting recipients are both suppressive

**Figure 6:**
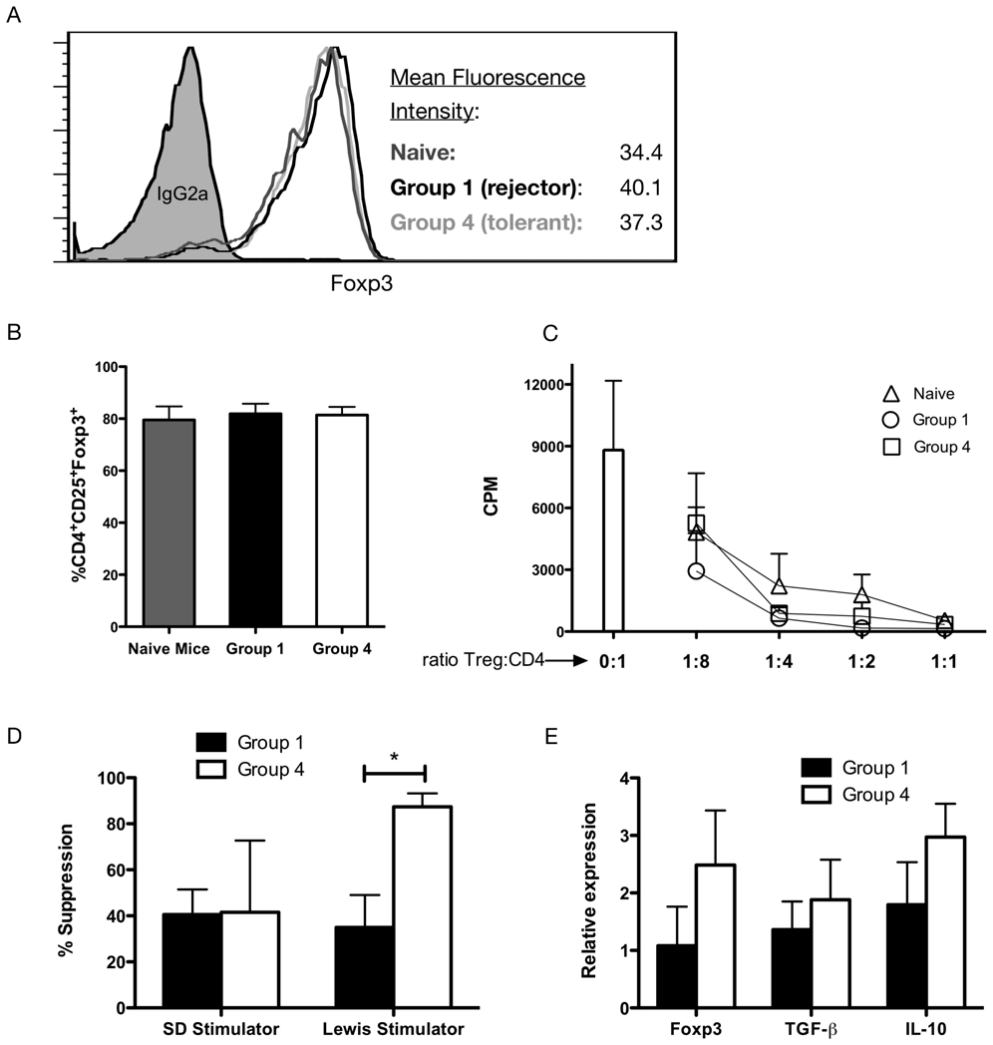
Functional characterization of Treg in rejecting and tolerant mice. Treg were isolated form the spleen of Group 1 (rejecting mice) at day 10 post transplantation before rejection occurrence and of Group 4 (tolerant mice) at day 20 after tolerance induction. Three mice per Group in 3 separate experiments showed similar mean fluorescence intensity (**A**) and purity (**B**) of Foxp3^+^ cells (gated on CD4^+^CD25^+^). Treg of Group 1 and 4 were used at different ratio (1∶1, 1∶2, 1∶4, and 1∶8) in co-culture with syngeneic responders (CD4^+^CD25^+^) and stimulators and anti CD3e mAb (**C**). Treg xenospecificity was assed by co-culture of naive mice splenocytes with donor Sprague Dawley or third party Lewis stimulators plus minus rejector or tolerant Treg. Percentage of suppression are shown (**D**). Foxp3, TGFβ-1 and IL-10 mRNA expression were measured by real time PCR. Results were calculated on basis of relative mRNA expression compared to naive mice (relative expression = 1, **E**).

To assess the functionality of Treg from rejecting recipients, tolerant mice, or naive mice, *in vitro* proliferation assays were performed (Figure 6). Treg were isolated from the spleen of untreated mice (Group 1) at day 10 before the occurrence of rejection and of Group 4 after tolerance induction at day 20. The purity and mean fluorescence intensity of Foxp3^+^ cells gated in CD4^+^CD25^+^ cells after isolation were comparable between the groups (Figure 6A fluorescence intensity and 6B purity of Treg in naive mice: 79.5%, in Group 1: 81.9% and in Group 4: 81.4%). Treg of Group 1 and 4 both revealed suppressive activity (Figure 6C). At a 1∶1 ratio (or Treg to CD4^+^CD25^−^ cells), Treg completely inhibited the proliferation of co-cultured CD4^+^CD25^−^ cells in response to polyclonal stimulation (syngeneic irradiated stimulators and anti-CD3 mAb). Interestingly Treg of tolerant and rejecting animals showed no xenospecificity in the in vitro xenogeneic suppression assays when compared with donor Sprague Dawley or third party Lewis stimulators (Figure 6D). In contrast, mRNA expression for Foxp3 was increased in Treg of tolerant recipients but not in rejector animals. TGF-β1 and IL-10 mRNA expression had a similar trend (Figure 6E).

## Discussion

Previous studies have demonstrated the induction of peripheral tolerance to allogeneic antigens in small animal models by a combined blockade of T cell costimulatory and proliferatory signals [2], [3]. In our model of islet xenotransplantation the application of RAPA and MR1 for 14 days post Tx allowed long-term survival of concordant islet xenografts over 100 days in the majority of recipients. These results suggested that peripheral tolerance was induced. However, as anti-CD154 mAbs are unlikely to be introduced into the clinic due to thromboembolic complications, the development of alternative strategies such as anti-CD40 mAbs should be evaluated in future studies.

The combined blockade of the co-stimulation (signal 2) and proliferation signaling (signal 3) with respectively MR1 and RAPA showed a strong and potent tolerogenic effect in our model (Figure 1). The ligation of CD40 by CD154 is crucial for activation and maturation of dendritic cells though upregulation of MHC and the costimulatory molecules CD80 and CD86, for activation and class switch of B cells and for the production of pro-inflammatory molecules such as TNF and IL-12 [19]. T cell activation is further inhibited through the anti-proliferative effect of RAPA. In addition, RAPA and MR1 have been described respectively to promote expansion of Treg and to enhance their levels of suppressive activities [20]–[23]. Consequently, the Treg suppressive activity may have potentiated the simultaneous blockade of signal 2 (co-stimulation) and signal 3 (proliferation) allowing development of long term graft tolerance. Therefore, the potential role of Treg in tolerance induction and maintenance was further investigated. In line with the concept of IL-2 dependency of Treg [24], [25], we used anti-IL-2 mAb (S4B6-1) and anti-CD25 mAb (PC61) to “starve” or to deplete Treg consistent with other reports using the same clones [26]–[28]. Administration of anti-IL-2 or anti-CD25 mAb at the time of islet Tx efficiently depleted Treg in the peripheral blood (Figure 2F, 3A) and was associated with a 90–100% rejection rate in MR1 and RAPA treated recipients. However, when these reagents were given 100 days post-Tx in tolerant animals, significantly less mice developed late rejection. This result suggests that Treg are essential during the induction phase of tolerance, i.e. immediately following concordant islet xenotransplantation under RAPA and MR1. Intriguingly, during the maintenance phase of tolerance, rejection is triggered by anti-IL2/anti-CD25 in some mice, suggesting a variable role for regulation at late time points post-transplant. This observation may be explained by a progressive decline in the role of Treg as deletion progresses over time. A similar process has been observed in other tolerance models [29]. Such a scenario is further supported by the increase in Treg numbers in the early phase post-transplant, but not in the late phase (Figure 3). Thus there will be a prolonged period during which both deletion and regulation are important. Depending on the specific degree of its contribution in individual mice at any given time, it would be expected that abrogation of regulation may lead to rejection or not.

The balance between effector T cells and Treg depends on the local microenvironment which seems to be an essential factor determining the fate of a graft [30]. No correlation was found between the percentage of Treg in blood and the fate of the xenograft. Indeed rejecting and tolerant mice had both higher percentages of Treg in the blood compared to baseline. In rejecting mice, Treg transiently increased in every compartment including the secondary lymphoid organs and the graft early during rejection (<48 h). A few days post-rejection, Treg disappeared in all compartments highlighting the importance of the time course post transplantation when Treg are analyzed. Contradictory studies have reported either an increase or a decrease of Treg in rejecting recipients [31]–[33]. Haanstra et al. transplanted allogeneic non-human primate kidneys and performed serial biopsies of these grafts over time showing infiltration of Treg in rejecting grafts. Wang et al. transplanted allogeneic mouse kidneys and found a reduction of Foxp3 expression in graft of rejecting mice. It is likely that these contradicting results can be due to different timing in assessment of Treg during the rejection process. In our study, suppression assays confirmed the functionality of Treg in rejecting recipient which was similar to Treg of tolerant mice. These results suggest that functional Treg develop during the process of rejection in response to xenoantigens, and that these cells disappear after the destruction of the islet xenograft.

In tolerant mice, Treg depletion early post-Tx resulted in rejection. These findings correlated with higher frequencies of Treg in the grafts and paLN at day 20 post-Tx, when compared to day 100 post Tx, which is consistent with the notion of draining lymph nodes and the graft itself as being primary sites for Treg function [9], [34]–[37]. Furthermore, the lack of an increase of Treg in the spleen is in line with other (allo-)tolerance models [38]. However, due to a lack of available markers the respective roles of induced and natural Treg in the early phase towards tolerance induction could not be examined. Thus, because induced and natural Treg may play different roles through different mechanisms during tolerance induction, it will be of interest to understand whether the observed increase of Treg in the draining lymph nodes and within the graft is the consequence of migrating natural Treg or of a local conversion of naive T cells to induced regulators. In our xenotransplantation model, Treg harvested from the spleen of tolerant animals expressed higher mRNA levels of Foxp3 suggesting a higher functional status, as reported recently [39]. However when tested *in vitro*, Treg of tolerant mice were neither more suppressive than Treg of rejector mice, nor xenospecific except when third party stimulators were used. These findings may be explained by the low frequency of donor specific-Treg induced in the spleen, despite the fact that RAPA/MR1 treatment has a non-specific systemic effect on Treg as shown by the increased levels of Foxp3. In summary, these results emphasize the importance of the compartmentalization of the xenogeneic immune response and its regulation.

In conclusion, administration of anti-IL-2 mAb or anti-CD25 mAb during the time of Tx prevented tolerance induction in our islet xenotransplantation model, suggesting that regulation by IL-2-dependent CD4^+^CD25^+^ T regulatory cells was critical in the induction of tolerance during the immediate post-Tx period. Delayed administration of anti-IL-2 mAb or anti-CD25 mAb did not abrogate tolerance in most recipients, indicating that maintenance of tolerance became less dependent on regulation over time, possibly indicating a role of progressive clonal deletion. These results were confirmed by the presence of Treg in paLN and grafts of tolerant mice early post-Tx but not after 100 days post-Tx suggesting a critical role of Treg for xenograft acceptance early after concordant islet xenotransplantation.

## Supporting Information

Table S1 Primer sequences used for real time PCR are listed in Table S1.
(0.03 MB DOC)

Material and Methods S1 (0.03 MB DOC)

Figure S1 *Concordant xeno- and allo-responses are diminished in late tolerant mice.* White bars, naïve mice; gray bars: Group 6, (late anti-IL2 treatment); black bars: Group 8 (late anti-CD25 mAb treatment). Alternatively Sprague donor strain splenocytes, Lewis splenocytes, BALB/c splenocytes or human PBMC were used as stimulators. In late anti-IL2 mAb and late anti-CD25 mAb treatment groups, graft-tolerant mice demonstrated MLR responses against donor cells that were reduced approximately 60% compared to control group and maintained a robust T cell proliferation against human stimulator cells. Surprisingly, graft-tolerant mice also showed significantly decreased T cell proliferation indices against allogeneic (Balb mouse) and xenogeneic (Lewis rat) stimulators compared to naïve mice. In rejecting mice, all mean stimulation indices were not statistically different when compared to naïve mice (data not shown). Late tolerant mice (200 days post tx) were shown to be hypo-responsive against donor antigen in contrast to rejecting or naive recipient in mixed lymphocyte reaction. Stimulation index was calculated as CPM of responder lymphocytes stimulated by allo- or xenogeneic stimulators divided by CPM of responder lymphocytes stimulated by self stimulators. All results were calculated with mean and standard deviation. One way ANOVA test and Bonferroni's multiple comparison post test was used. * P<0.05, ** P<0.01 and ***P<0.001 were considered significant.
(4.77 MB TIF)

Figure S2 *Humoral response and complement deposition analysis of rejecting and tolerant mice.* Immunohistology for humoral immune responses to concordant islet xenografts in untreated C57BL/6 mice and in combination therapy (MR1+RAPA) treated mice at 200 days post transplantation. Sections were stained by anti-mouse IgG (A/D), IgM (B/E) and complement (C3, C/F). A humoral response was detected at rejection with the presence of IgG (A), IgM (B), and C3 (C), whereas neither immunoglobulin (IgG, D, and IgM, E,) nor complement deposition (F) was observed in tolerant grafts. (Magnification IgG (100x), IgM (100x), C3 (100x)).
(6.70 MB TIF)
