# Supplementary material for: Anti-CD154 mAb and Rapamycin Induce T Regulatory Cell Mediated Tolerance in Rat-to-Mouse Islet Transplantation
Source: PLoS One. 2010 Apr 26;5(4):e10352. doi: 10.1371/journal.pone.0010352 (PMC2859949; doi:10.1371/journal.pone.0010352)
Supplement: Table S1 — Primer sequences used for real time PCR are listed in Table S1. (0.03 MB DOC) [file pone.0010352.s001.doc]

Supplementary Table 1

| Gene | Forward primer | Reverse primer |
| --- | --- | --- |
| *Foxp3* | 5’-TCACCTATGCCACCCTTATC-3’ | 5’-AGGCGAACATGCGAGTAAAC-3’ |
| *IL-10* | 5-GAATTCCCTGGGTGAGAAGC-3’ | 5’-CTCTTCACCTGCTCCACTGC-3’ |
| *TGF-beta1* | 5’-AGGTCACCCGCGTGCTAATG-3’ | 5’-TCTGCACGGGACAGCAATGG-3’ |
| *GAPDH* | 5’-CCCGTAGACAAAATGGTGAAG-3’ | 5’-AGGTCAATGAAGGGGTCGTTG-3’ |
