# Supplementary material for: Anti-CD154 mAb and Rapamycin Induce T Regulatory Cell Mediated Tolerance in Rat-to-Mouse Islet Transplantation
Source: PLoS One. 2010 Apr 26;5(4):e10352. doi: 10.1371/journal.pone.0010352 (PMC2859949; doi:10.1371/journal.pone.0010352)
Supplement: Material and Methods S1 — (0.03 MB DOC) [file pone.0010352.s002.doc]

*Supporting Material and Methods 1*

Mixed lymphocyte reaction (MLR)

One way mixed lymphocyte reaction (MLR) was performed for mice transplanted with SD rat islets in Group 6 (late anti-IL2 mAb treatment) and in Group 8 (late anti-CD25 mAb) treatment groups at day 200 post islet Tx. As control, non-transplanted naïve C57BL/6 mice were also used as responders.

Briefly mouse and rat mononuclear cells were harvested from the spleen as previously described [12]. Mononuclear cells harvested from non-transplanted C57BL/6 and transplanted mice of Groups mentioned above were designated as responder cells.  Mononuclear cells derived from naïve C57BL/6 and BALB/c male mice, Sprague-Dawley and Lewis male rats were designated as stimulator cells respectively. Stimulator human mononuclear cells were prepared from buffy coat obtained from the blood bank of the University Hospital of Geneva. All cells were purified by Ficoll/Histopaque (Sigma, Buchs, Switzerland) gradient centrifugation. Stimulator cells were irradiated using a γ irradiator with 3500 Rad.

For proliferation assays, stimulator cells were cultured in a 2:1 ratio with responder cells in medium, i.e. 4x10^5^ responder cells were cultured with 8x10^5^ irradiated stimulator cells in 96-well round-bottom plates (Nunclon™ Surface, DK-4000 Roskilde, Danmark). Four separate wells were dedicated to each responder-stimulator combination and each experiment was repeated three times. Cells were incubated for 5 days at 37°C in 95% humidified air mixed with 5% carbon dioxide. On day 5, 2μCi^3^[H]-thymidine was added to each well. The incorporation of ^3^[H]-thymidine was assessed as count per minute (CPM). Degree of T-cell proliferation was interpreted using a stimulation index (SI). This stimulation index was calculated as follows: CPM of responder lymphocytes stimulated by allo- or xenogeneic stimulators divided by the CPM of responder lymphocytes stimulated by self-isogeneic stimulators.
