# Supplementary figures and images for: Anti-CD154 mAb and Rapamycin Induce T Regulatory Cell Mediated Tolerance in Rat-to-Mouse Islet Transplantation
Source: PLoS One. 2010 Apr 26;5(4):e10352. doi: 10.1371/journal.pone.0010352 (PMC2859949; doi:10.1371/journal.pone.0010352)

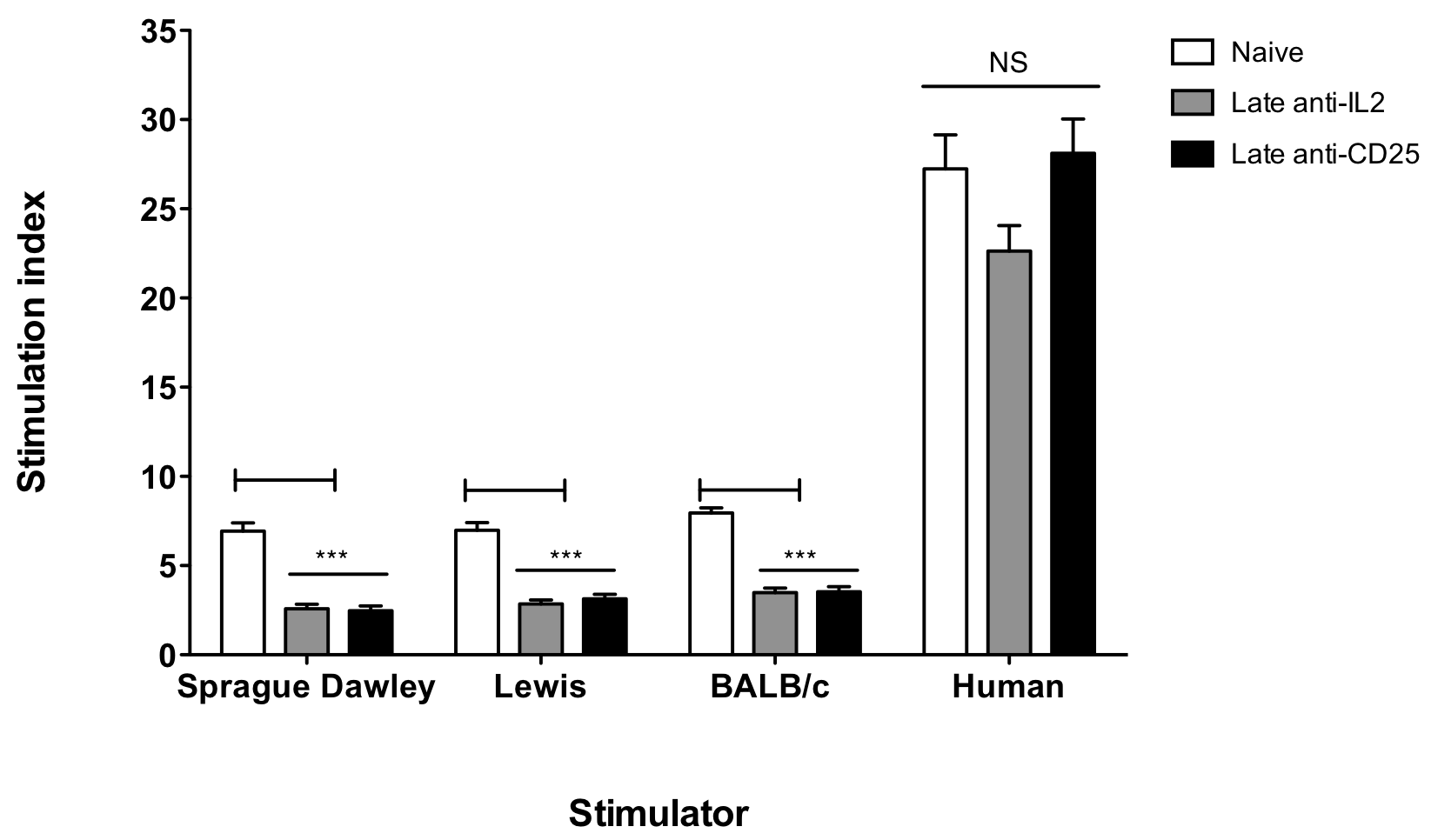

Supplement: Figure S1 — Concordant xeno- and allo-responses are diminished in late tolerant mice. White bars, naïve mice; gray bars: Group 6, (late anti-IL2 treatment); black bars: Group 8 (late anti-CD25 mAb treatment). Alternatively Sprague donor strain splenocytes, Lewis splenocytes, BALB/c splenocytes or human PBMC were used as stimulators. In late anti-IL2 mAb and late anti-CD25 mAb treatment groups, graft-tolerant mice demonstrated MLR responses against donor cells that were reduced approximately 60% compared to control group and maintained a robust T cell proliferation against human stimulator cells. Surprisingly, graft-tolerant mice also showed significantly decreased T cell proliferation indices against allogeneic (Balb mouse) and xenogeneic (Lewis rat) stimulators compared to naïve mice. In rejecting mice, all mean stimulation indices were not statistically different when compared to naïve mice (data not shown). Late tolerant mice (200 days post tx) were shown to be hypo-responsive against donor antigen in contrast to rejecting or naive recipient in mixed lymphocyte reaction. Stimulation index was calculated as CPM of responder lymphocytes stimulated by allo- or xenogeneic stimulators divided by CPM of responder lymphocytes stimulated by self stimulators. All results were calculated with mean and standard deviation. One way ANOVA test and Bonferroni's multiple comparison post test was used. * P<0.05, ** P<0.01 and ***P<0.001 were considered significant. (4.77 MB TIF) [file pone.0010352.s003.tif]

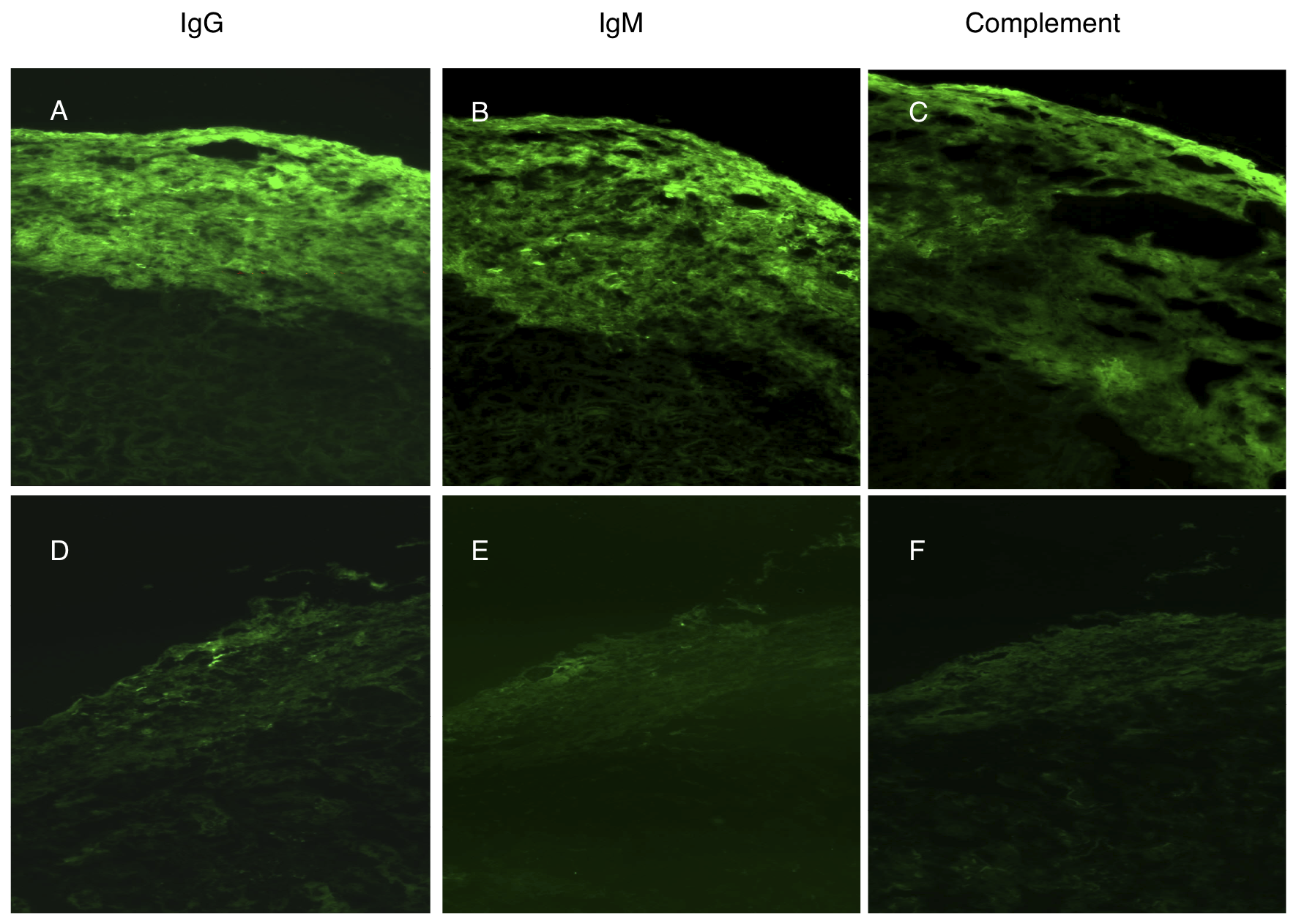

Supplement: Figure S2 — Humoral response and complement deposition analysis of rejecting and tolerant mice. Immunohistology for humoral immune responses to concordant islet xenografts in untreated C57BL/6 mice and in combination therapy (MR1+RAPA) treated mice at 200 days post transplantation. Sections were stained by anti-mouse IgG (A/D), IgM (B/E) and complement (C3, C/F). A humoral response was detected at rejection with the presence of IgG (A), IgM (B), and C3 (C), whereas neither immunoglobulin (IgG, D, and IgM, E,) nor complement deposition (F) was observed in tolerant grafts. (Magnification IgG (100x), IgM (100x), C3 (100x)). (6.70 MB TIF) [file pone.0010352.s004.tif]
